# Changes in mean platelet volume and hematologic indices in patients with panic disorder due to oxidative stress

**DOI:** 10.1002/brb3.1569

**Published:** 2020-02-25

**Authors:** Maryam Naghipour Hamzekolaei, Moslem Jafarisani, Asghar Farajzadeh, Seyed Shahrokh Aghayan, Amir Atashi, Maryam Yarmohammadi, Iman Sadeghi, Mersedeh Tashakori

**Affiliations:** ^1^ Veterinary School Orumiyeh University Orumiyeh Iran; ^2^ Clinical Biochemistry School of Medicine Shahroud University of Medical Sciences Shahroud Iran; ^3^ Department of Clinical Laboratory Sciences Ardabil Branch Islamic Azad University Ardabil Iran; ^4^ School of Medicine Shahroud University of Medical Sciences Shahroud Iran; ^5^ Department of Hematology School of Allied Medicine Shahroud University of Medical Sciences Shahroud Iran; ^6^ Genetic, Ceinge Biotechnologia Avanzate Napl Italy; ^7^ Department of Biology, Science and Research Branch Islamic Azad University Tehran Iran

**Keywords:** MCHC, MPV, oxidative stress, panic disorder, RDW

## Abstract

**Objective:**

Cardiovascular disorders are common in patients with panic disorder (PD), usually mediated by platelets. The present study was conducted to evaluate oxidative stress conditions and complete analysis of blood cells in patients with PD.

**Setting and Sample Population:**

Sixty healthy individuals and 60 patients were included in the study. Whole blood and serum samples were obtained from patients and controls.

**Materials & Method:**

Hematological studies, including blood cells count, hemoglobin, and hematocrit, were carried out on whole blood samples. In addition, oxidative stress indices including total antioxidant capacity, free oxygen species, and malondialdehyde concentration were measured in serum samples.

**Results:**

Results showed that patients with PD had a significant increase in mean platelet volume index (MPV), platelet distribution width (PDW), red blood cell distribution width (RDW), and mean corpuscular hemoglobin concentration (MCHC) compared with healthy subjects (*p* < .05). Also, oxidative stress indices were significantly elevated in patients with PD compared with control group (*p* < .05).

**Conclusion:**

Elevated MPV is a hematologic indicator for patients with PD. This disorder may be caused by impaired serotonin metabolism, resulting in increased oxidative stress, as well as in platelet serotonin transporters. Regarding elevated oxidative stress, the risk of cardiovascular complications is high in patients with PD.

## Graphical abstract

Panic patients have changed in MPV in relation to serotonin metabolism. It is because of oxidative stress due to serotonin metabolism.

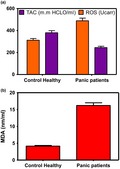

**Significant outcome**

- These data in patients with PD highlight an altered oxidative stress due to serotonin metabolism.
- MPV, PDW, RDW, and MCHC promise as biomarkers for cardiovascular disorder in patients with PD.

**Limitation**

- The study may not have enough power to detect serotonin metabolites.
- The lack of validation experiments is a caveat.

## 1 INTRODUCTION

Panic disorder (PD) is one of the most common serious disorders in the field of psychological illness characterized by sudden and unexpected attacks (Freire, Zugliani, Garcia, & Nardi, 2016). Symptoms of a panic attack include palpitations, sweating, shaking, shortness of breath, and fear of losing control (APA, 2000). Serotonergic system disorders and their receptors are thought to be effective in the neurobiology of the disease (Maron & Shlik, 2006). Although clinical and experimental researches, brain scans, and genetic and drug studies have confirmed this issue, but two hypotheses that justify it are contradictory. First hypothesis implies elevated serotonin level or activity whereas second hypothesis refers to decreased serotonin level or activity. The first hypothesis seems to be stronger because of numerous studies (Bell & Nutt, 1998; Iversen, 1984).

Serotonin is a derivative of tryptophan that is produced in the nervous system at the terminus of serotonergic neurons in the gastrointestinal tract. Also, serotonin is stored in platelet granules during their production. The role of serotonin in the platelet activity and aggregation has been established. Serotonin transporters in platelets and neurons are quite similar, and platelets are considered as a suitable storage source for serotonin. In addition, studies have shown that the levels of serotonin storage in cerebrospinal fluid and platelets are correlated (Audhya, Adams, & Johansen, 2012; Mercado & Kilic, 2010).

Platelets are taught to be a good tool to reflect metabolic disorders in the nervous system. In order to evaluate platelet functions, there are several laboratory parameters including platelet count (Plt), mean platelet volume index (MPV), and platelet distribution width (PDW), which latter is more suitable (Camacho & Dimsdale, 2000; Huczek et al., 2005). Studies have linked the increase in MPV index with cardiovascular disease such as myocardial infarction and ischemia. Researchers have suggested that elevated MPV is an indicator of 6‐month mortality risk for heart failure patients (Canan et al., 2012; Stratz et al., 2008).

Studies have shown that platelets in peripheral blood reflect serotonin metabolism in the nervous system and hence the function of serotonergic neurons. Serotonin is an important factor in the pathophysiology of neurodegenerative diseases such as anxiety and plays an important role in platelet aggregation (Camacho & Dimsdale, 2000).

Platelets at their surface express a variety of serotonin receptors, including 5‐HT2A and 5HT3. Serotonin transporter expression has also been reported in platelet membranes. Studies have shown that serotonin as a procoagulant is effective in thrombosis. These effects are usually mediated by serotonin reuptake inhibitor (SSRI) drugs used in patients with PD (Galan et al., 2009; Markovitz, Shuster, Chitwood, May, & Tolbert, 2000; Purselle & Nemeroff, 2003).

Various abnormalities of serotonin receptors have been identified in patients with PD, and it has been concluded that increased serotonin production occurs as a result of serotonin transporter dysfunction, leading to anxiety and fear in response to amygdala stimulation (Ataoglu & Canan, 2009; Gogcegoz Gul, Eryilmaz, Ozten, & Hizli Sayar, 2014). Conversely, serotonergic agents are used to relieve symptoms of PD. As serotonin levels increase, its catabolism will definitely increase and studies have reported increased activity of monoamine oxidase (MAO) activity in cerebrospinal fluid (Karacetin et al., 2012; Martins et al., 2019; Mathew, Srinivasan, Johnson, Thomas, & Mandal, 2019; Sahin, Turan, Neselioglu, Can, & Atagun, 2019).

As a result, the activity of oxidase enzymes induces the process of producing free radicals which can elevate oxidative stress production. Increased oxidative stress elevates the risk of cardiovascular disease directly and also indirectly through the activation of platelets to produce thrombosis (Freedman, 2008).

Imbalance between oxidant and antioxidant called as oxidative stress. We can assess the oxidative stress by assessment of some indexes such as malondialdehyde (MDA), reactive oxygen species (ROS), and total antioxidant capacity (TAC) in serum of patients. Oxidative stress can cause many disorders such as damage to RBC, damage to DNA and proteins, alteration structure of LDL, and produced oxidized LDL. It elevates risk of cardiovascular disease (Amin et al., 2020; Flora, 2020).

The aim of the present study was to survey the changes in platelet and hematologic indices and their relation with oxidative stress in patients with PD in comparison with healthy subjects.

## 2 METHODS

The present case–control study was conducted for 9 months in a university‐affiliated clinic under the code of ethics IR.SHMU.REC.1397.90 at the Shahroud University of Medical Sciences. Sixty healthy subjects and sixty patients diagnosed by a neurologist were included in the study. The age range of the subjects was 18–45 years. The study excluded patients with history of neurological disease, tobacco use, or any chronic illness.

All the participants signed the informed written consent and completed a demographic questionnaire. In addition, 5 cc venous blood samples were collected in an EDTA tube to get whole blood and a tube without anticoagulant to get serum. Hematologic tests were analyzed by Sysmex K21, and Plt, MPV, PDW, hemoglobin, and hematocrit were measured. In addition, oxidative stress indices including MDA, TAC, and ROS were measured using commercial kits (Razi Technologies) according to company guidelines. Quantitative results were reported based on mean and standard deviation. Statistical analysis was performed by two‐way independent *t* test and Fisher's exact test, and *p* ≤ .05 was considered significant.

## 3 RESULTS

The demographic survey of the present study showed that the mean age of the study population was 35.28 ± 10.23 which 37% of them were men (Table 1). Although more females participated in present study, there was no significant relationship between sex and disease.

**Table 1:** Demographic characteristics of panic disorder patients and healthy individuals

Variable	Standard deviation ± Mean		*p* value
	Healthy individuals (60)	Panic disorder patients (60)	
Age (year)	35.28 ± 10.23	33.42 ± 12.63	*t* = 0.45, *df* = 263, *p* = .67
Gender (%)			
Male	22 (37)	29 (48)	*p* = .41
Female	38 (63)	31 (52)	

Note

Based on nonpaired *t* test and the Fisher test, no significant correlation was found.

As it shown in Table 2, the indices, including RBC, Plt, mean corpuscular hemoglobin concentration (MCHC), red blood cell distribution width (RDW), MPV, and PDW, were significantly different in the patients compared with the healthy individuals. Individuals with PD had lower levels of RBC compared with healthy group.

**Table 2:** Hematologic indices in patients with panic disorder and healthy subjects

Variable	Standard deviation ± Mean		The significance level (*p*, *df* = 248)
	Panic disorder patients (60)	Healthy individuals (60)	
Hb (g/dl)	12.46 ± 1.32	13.36 ± 2.0	*t* = 1.87, *p* = .09
RBC (×10^12^/L)	4.01 ± 0.73	4.9 ± 0.93	*t* = 3.87, *p* = .05
WBC (×10^9^/L)	7.3 ± 0.42	8.32 ± 1.4	*t* = 2.57, *p* = .07
Plt (×10^12^/L)	245.18 ± 65.2	285.48 ± 47.2	*t* = 3.64, *p* = .004
MCV (fl)	81.27 ± 8.32	83.17 ± 7.54	*t* = 1.91, *p* = .31
MCH (pg)	28.1 ± 3.2	28.43 ± 2.32	*t* = 2.37, *p* = .29
MCHC (g/L)	32.75 ± 1.43	34.65 ± 2.1	*t* = 7.58, *p* ≤ .0001
RDW (fl)	17.38 ± 2.65	15.28 ± 2.39	*t* = 6.29, *p* ≤ .0001
MPV (fl)	6.98 ± 0.64	8.53 ± 1.1	*t* = 10.43, *p* ≤ .0001
PDW (fl)	17.3 ± 1.01	14.73 ± 2.84	*t* = 11.87, *p* ≤ .0001

Abbreviations: MCH, mean corpuscular hemoglobin or mean cell hemoglobin; MCHC, mean corpuscular hemoglobin concentration; MCV, mean corpuscular volume; MPV, mean platelet volume index; PDW, platelet distribution width; RDW, red blood cell distribution width.

**Figure 1:**
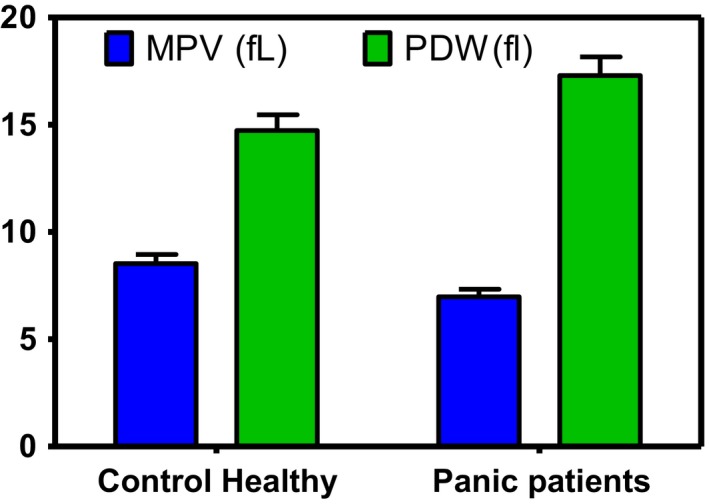
Mean platelet volume index (MPV) and platelet distribution width (PDW) values in healthy individuals and panic disorder patients. ANOVA analysis showed a significant deference in MPV and PDW in panic patients in comparison with healthy control. (*p* < .03)

**Figure 2:**
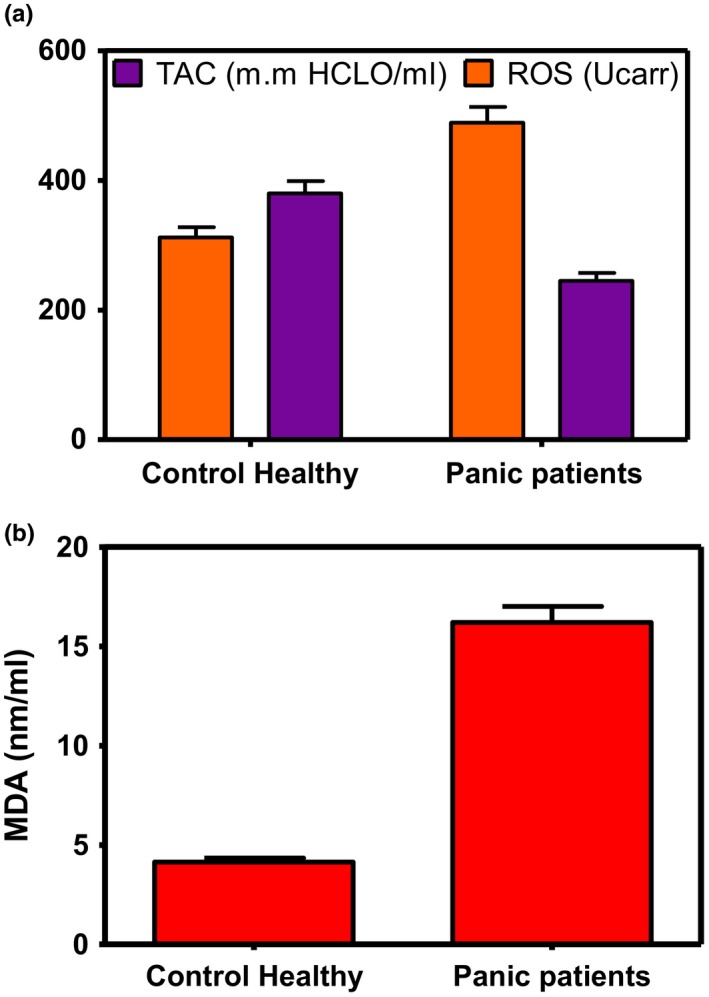
Malondialdehyde (MDA), reactive oxygen species (ROS), and total antioxidant capacity (TAC) values in healthy individuals and panic disorder patients. (a) According to the ANOVA analysis, our results showed a significant increase of ROS and decrease of TAC in panic patients in comparison with healthy individuals (*p* < .03). (b) According to the ANOVA analysis, our results showed a significant increase of MDA in panic patients in comparison with healthy individuals (*p* < .03)

In addition, according to Figure 1, Plt and MPV were significantly decreased in patients. On the other hand, RDW and PDW values were significantly higher in the patients group compared with healthy individuals. As Figure 2 shows, we found significantly decreased TAC and increased ROS and MDA in patients with PD compared with control group.

## 4 DISCUSSION

The present study was designed to evaluate oxidative stress conditions and changes in platelet and hematologic indices in patients with PD. The current study found that oxidative stress is elevated in patients with PD, which was accompanied with changes in MPV, PDW, and RDW.

Since CSF collection is more invasive and difficult compared with plasma, evaluating peripheral blood platelets is used to investigate serotonin metabolism. Platelets are also used to monitor cellular signaling in neurodegenerative diseases as well as the serotonergic system (Camacho & Dimsdale, 2000). Although the platelet maturation process in bone marrow has been identified as an influencing factor on MPV, there is some evidence suggesting age and heterogeneity effect on MPV (May, Marques, Reddy, & Gangaraju, 2019).

The results of this study showed that people with PD experience higher levels of oxidative stress and lower MPV and PDW values compared with healthy subjects. These findings are in line with the findings of some researchers, but are contradictory with Gogcegoz Gul observations, who reported an increase in MPV (Gogcegoz Gul et al., 2014). There has been little research on the association between MPV and psychological illnesses. However, Ataoglu et al. reported higher levels of MPV in patients with acute depression compared with healthy individuals (Ataoglu & Canan, 2009).

Hoausberg et al. reported sympathetic overactivity in patients with major depressive disorder. Another study found the association of sympathetic activity with MPV (Hausberg, Hillebrand, & Kisters, 2007). Regarding the development of laboratory methods in the clinical hematology sciences, evaluation of parameters such as MPV, RDW, and PDW have become routine. However, they are not commonly used in diagnosis and monitoring (May et al., 2019). There have been several studies showing the relationship between platelets count and serotonin transporters, which have subsequently been associated with an increased risk of cardiovascular disease in people with depression and anxiety (Vizioli, Muscari, & Muscari, 2009).

As discussed above, platelet volume depends on several factors, including increased platelets activation or production and presence of immature platelets in the peripheral blood. Platelet volume depletion occurs due to platelet activation and its discharge from the granules. Since platelet activation and consumption are associated with a decrease in peripheral blood Plt, therefore, bone marrow activity and the presence of immature platelets in the blood are inevitable, leading to an increase in PDW (Bayraktar & Albayrak, 2017; Tang et al., 2017). Studies of platelet disorders in people with anxiety indicate defects in the serotonin transporter. Studies by Martini et al. have shown that defects in platelet signaling and activation are associated with ERK1,2 phosphorylation. They suggested that elevated levels of serotonin receptors phosphorylation and carriers are responsible for the platelet disorder (Martini et al., 2004).

In the present study, a significant decrease in MPV indicates impaired metabolism of serotonin and its transporters, which could also be a therapeutic guide in these patients, as lithium users are characterized by improvements in this parameter (Atagün et al., 2016).

On the other hand, PDW is strongly affected by platelet activity and MPV. Platelet volume increases with platelet activation and decreases with platelet discharge. Also, during peripheral platelet consumption and increase in bone marrow activity immature platelets appear in peripheral blood, which is a cause of increased RDW (Nishimura et al., 2018; Tang et al., 2017).

Studies have shown that increased serotonin uptake and oxidative stress could be the cause of platelet activation. Increased serotonin metabolism and subsequent MAO activity induce oxidative stress, and consequently, oxidative stress may activate platelet activation (Bianchi, Pimentel, Murphy, Colucci, & Parini, 2005; Mialet‐Perez, Santin, & Parini, 2018; Sugiura et al., 2016). Our result showed a significant elevation of ROS in patient with PD and reduction in TAC, so it can affect the RBC membrane and causes significant alterations of RDW and MCHC (Mehrpour et al., 2018).

In this regard, we found significant increase of MDA in patients with PD compared with healthy patients. MDA as a marker of lipid peroxidation due to oxidative stress can be helpful to determine the oxidative stress status in patients with PD (Amin et al., 2020).

Also, the number of platelets decreased significantly in the present study, indicating that activated platelets were removed from the peripheral circulation or replaced in the vascular lesions and therefore decreased in the collected specimens. This may be the reason why the bone marrow is activated and immature platelets are appeared in the blood and PDW are elevated (Asoglu et al., 2016; Kokacya et al., 2015).

The present study found a decreased trend of RBC levels in patients with PD, statistically not significant, which is probably due to increased oxidative stress and could be justified by increased RDW in these patients, as elevated RDW has been reported in depression patients (Asoglu et al., 2016; Bhad, 2017; Ransing, Patil, & Grigo, 2017). On the other hand, these findings were accompanied with a significant decrease in MCHC, compatible with other studies, which suggests inflammation in patients with PD, as a possible cause of elevated RDW and PDW (Huang & Hu, 2016; McClung & Karl, 2009; Vogelzangs, Beekman, de Jonge, & Penninx, 2013). Totally, this study showed, because of impairment of serotonin metabolism in patients with PD, they showed elevated level of oxidative stress. So it can affect membrane of RBC and PLT. Due to the defect in antioxidant defense system, ROS can damage endothelial and activate Plt which are causes of cardiovascular disease (Heidenreich & Roth, 2020).

## 5 CONCLUSION

The present study showed that platelet activation in patients with PD is an inevitable complication. In addition, increased PDW and RDW indicate that the inflammatory system is active in these patients. Therefore, due to elevated oxidative stress and activated inflammatory system, these patients are at high risk of developing cardiovascular disease.

## CONFLICT OF INTEREST

None declared.

## AUTHOR'S CONTRIBUTION

Maryam Naghipour Hamzekolaei contributed to conceptualization, data curation, and investigation; Mersedeh Tashakori contributed to investigation; Asghar Farajzadeh contributed to software facilitation; Seyed Shahrokh Aghayan contributed to methodology; Amir Atashi contributed to formal analysis; Maryam Yarmohammadi contributed to validation and visualization; Iman Sadeghi contributed to resource facilitation; Moslem Jafarisani contributed to project administration, writing–original draft, writing–review editing, and supervision.

## Data Availability

The data that support the findings of this study are available from the corresponding author upon reasonable request.
